# CORRELATION OF SARS-COV-2 EXPRESSION IN THE PLACENTA AND THE INCIDENCE OF PREECLAMPSIA

**DOI:** 10.21010/Ajidv17i1.4

**Published:** 2022-12-22

**Authors:** LUMBANRAJA Sarma, EFFENDI Iman Helmy, SIAHAAN Andre M, BANCIN Berry E P, EDIANTO Deri, ASROEL Edwin Martin, SUDEWO Yudha

**Affiliations:** 1 Obstetrics and Gynecology Department, Faculty of Medicine, Universitas Sumatera Utara, Medan, 20136, Indonesia; 2 Neurosurgery Department, Faculty of Medicine, Universitas Sumatera Utara, Medan, 20136, Indonesia

**Keywords:** Preeclampsia, SAR-CoV-2, IHC, Nucleocapsid, Histopathology

## Abstract

**Background:**

The pandemic caused by SARS-CoV-2 also caused infection in some pregnant women. Some reports say this viral infection can show symptoms of preeclampsia.

**Material and Methods:**

We analyzed 25 pregnant women with SARS-CoV-2 infection with 4 patients presenting with symptoms of preeclampsia. we performed routine blood analysis, renal function, liver function, and IHC examination to see the expression of viral proteins in the placenta.

**Results:**

we obtained 4 patients with confirmed SARS-CoV-2 infection by RT-PCR. In these 4 cases, none of the cases showed expression of the SARS-CoV-2 viral protein in the placenta, and all 4 mothers were declared dead after treatment, and 2 babies delivered out of these 4 cases died. In one case we had fetal death in pregnancy while in one case prematurity. 2 babies born to mothers with SARS-CoV-2 infection with preeclampsia were born in good condition. There were no babies infected with SARS-CoV-2

**Conclusion:**

We conclude that SAR-CoV-2 infection in pregnant women with comorbidities can lead to a poor prognosis for both mother and baby. We cannot yet conclude whether SARS-CoV-2 infection can cause preeclampsia, but SARS-CoV-2 infection can exacerbate preeclampsia symptoms.

## Introduction

The SARS-CoV-2 outbreak was the first pandemic of this century. SARS-CoV-2 infection is transmitted through droplets; other transmission pathways are widely hypothesized but have yet to be confirmed. So far it is not clear whether and how SARS-CoV-2 can be transmitted from mother to fetus. We demonstrated transplacental transmission of SARS-CoV-2 in neonates born to mothers who were infected in the last trimester and exhibited neurological impairment. Its transmission was confirmed by comprehensive virological and pathological investigations(Fahmi *et al.*, 2021). Specifically, SARS-CoV-2 causes: (1) maternal viremia, (2) placental infection and very high viral load; inflammation of the placenta is demonstrated by histologic and immunohistochemical examination, and (3) neonatal viremia after placental infection. Neonates are studied clinically, through imaging, and followed up. Neonates present with neurologic manifestations, like those described in adult patients (Khan *et al.*, 2020).

Although SARS-CoV-2 infection has spread rapidly around the world, data on the natural history of infection in pregnant women and the risk of mother-to-child transmission are scarce. The physiological and immunological changes that occur as a normal component of pregnancy can have systemic effects that increase the risk of complications from viral infections. Recent data suggest that maternal blood levels of viral RNA are low and there is no evidence of placental infection with SARS-CoV-2. Reports published to date suggest that perinatal transmission of SARS-CoV-2 is possible but rare. Among 179 newborns tested for SARS-CoV-2 at birth to mothers with SARS-CoV-2, the transmission was suspected in 8 cases, 5 with positive nasopharyngeal SARS-CoV-2 RT-PCR and 3 with SARS-CoV-2 IgM. However, these cases arose from maternal infection before delivery and there is no information on exposition during the first or second trimester of pregnancy. Well-designed prospective cohort studies with rigorous assessment criteria are needed to determine the incidence and risk factors for perinatal SARS-CoV-2 transmission (Baergen & Heller, 2020).

During primary infection, entry of the virus into the blood, even for a short time, is an important prerequisite for maternal-fetal transmission to occur via the transplacental route. In the previous epidemic of a severe acute respiratory syndrome associated with Sars-CoV-1, ±78% of patients had detectable viral RNA in the blood within a week of the onset of symptoms (Chen *et al.*, 2020). Viremia was determined using a quantitative PCR assay specific to the Sars-Cov-1 genome, with a detection limit of 74 copies/ml in plasma (Fahmi *et al.*, 2021; Gujski *et al.*, 2020). The plasma viral load found in patients with so-called “moderate” symptoms was low with a mean concentration of 140 copies/mL, close to the detection threshold. This SARS-1 study was carried out with the optimized method, while the optimization of the method for detecting viremia is still ongoing for the SARS-CoV-2 study. In patients with SARS-CoV-2, the SARS-CoV-2 virus may not be detected by the test PCR on oropharyngeal samples. In two cohort studies of 205 and 40 patients, the presence of plasma viral RNA was detected in only 1% and 15% of patients, respectively(Chen *et al.*, 2020; Khan *et al.*, 2020; Kotlyar *et al.*, 2021).

The data obtained from several studies regarding the transplacental infection of SARS-CoV-2 from mother to baby have not shown satisfactory results. The existing reports are only in the form of case reports, and the developed treatments have not given good results. This is certainly a complex problem because it contributes to an increase in maternal and infant mortality rates in Indonesia, so research is needed on this matter to minimize the adverse effects that may arise in the future. So, the authors are interested in researching the mechanism of transmission of SARS-CoV-2 from mother to baby and knowing the effectiveness of COVID screening and the description of the characteristics of pregnant women with SARS-CoV-2 infection at Haji Adam Malik Hospital in Medan.

## Materials and Methods

### Ethics statement

This research has been approved by the Health Research Ethics Committee, Faculty of Medicine, Universitas Sumatera Utara (approval number: 92/KEP/USU/2021) dated March 9^th^, 2021.

### Population and sampling

We enrolled 28 pregnant women with confirmed SARS-CoV-2 infection by RT-PCR. At the time of termination, we took a sample of the placenta and performed an immunohistochemical examination to determine the N protein expression of SARS-CoV-2 in the placenta. Characteristic data we get through the patient’s medical record. We also perform complete blood counts, kidney function tests, liver function tests, urinalysis, and chest X-rays, as well as perform RT-PCR examinations and serologic examinations of the umbilical cord in newborn babies.

### Placental IHC examination

Collection of clinical data and surgical tissue paraffin blocks that have met the inclusion criteria, Clinical data recording, Paraffin block cutting, and preparation of H&E smear preparations. The H&E preparations were evaluated by the researcher and two specialists in anatomic pathology. Serial cutting of paraffin blocks for use as preparations which will then be stained with immunohistochemical staining using rabbit antibody-antigen SARS-CoV-2 (COVID-19) nucleocapsid antibody [HL344] (GeneTex)) with a dilution of 1: 200 which will appear positive in the nucleus; and the cytoplasm of the cytotrophoblast and syncytiotrophoblast cells. The positive control used was tissue from the patient’s lung biopsy. Assessment of the immunohistochemical expression of N protein was done semi-quantitatively by the researcher and two specialists in anatomic pathology. The data obtained will be tabulated and a statistical assessment will be carried out to determine the characteristics of the sample and assess the immunohistochemical expression of N protein (nucleocapsid) in each placental image from pregnant women with COVID-19 infection.

### IHC interpretation

The interpretation of the immunohistochemical description in this study used the Allred score, which is a semi-quantitative scoring system consisting of two assessments, namely the proportion score and the intensity score. The proportion score describes the percentage of cells stained by antibodies while the intensity score describes the strength of the staining of cells by antibodies.

**Figure 1:**
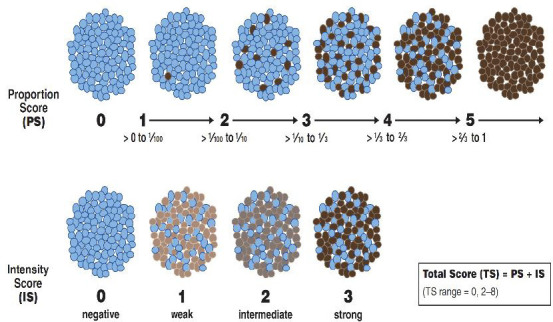
Intensity and Proportion Scores.

## Statistical analysis

Data will be presented in the form of categories, nominal, and ordinal data. The data is then grouped based on the data group and a normality test is performed on the numerical data. Data that is normally distributed will be presented in the form of mean ± standard deviation, while data that is not normally distributed will be presented in the form of mode (min-max), and nominal data will be presented in the form of n (%). Data were analyzed using chi-square or fisher’s exact test on categorical data, while numerical were analyzed using the T-test on normally distributed data, while data that were not normally distributed were analyzed using the Mann Whitney u test.

### Data availability

### Underlying data

Mendeley Data: N protein SARS-CoV-2 placental expression. https://data.mendeley.com/v1/datasets/dsk6xtwr7s/draft?preview=1

Data are available under the terms of the Creative Commons Zero “No rights reserved” data waiver (CC0 1.0 Public domain dedication).

## Results

In this study, we obtained 28 patients who met the criteria for inclusion in the study. Twenty-eight patients involved in this study came to Haji Adam Malik General Hospital and underwent RT-PCR examination, the gold standard for diagnosis of COVID-19. The characteristics of the patients involved in this study can be seen in Table 1.

**Table 1:** Sample Characteristics

Variable	
Age, years (mean±SD)	29 ± 6
Body weight, kg (mean±SD)	56 ± 4
Height, cm (mean±SD)	157 ± 5
Body mass index, kg/m2(mean±SD)	22.6 ± 1.7
Upper arm circumference, cm (mean±SD)	26.7 ± 1.2
Gravida (mean±SD)	2 ± 1
Parity (mean±SD)	1 ± 1
Abortion (mean±SD)	0 ± 1
Gestational age at termination, weeks (mean±SD)	36 ± 1
SARS-CoV-2 antigen (n, %)	
Not Reactive	1 (3.6)
reactive	27 (96.4)
Hb, g/dl (mean±SD)	11.7 ± 2.1
HT, % (mean±SD)	35 ± 6
Leukocytes, /μL (mean±SD)	12800 ± 7128
Platelets, /μL (mean±SD)	274750 ± 104752
Absolute Neutrophils, % (mean±SD)	10.35 ± 6.41
Absolute Lymphocytes, % (mean±SD)	1.5 ± 0.83
D-Dimer, L /ml (mean±SD)	1447 ± 1072
Fibrinogen, mg/dl (mean±SD)	554 ± 182
SGOT (mean±SD)	23.68 ± 8.11
SGPT (mean±SD)	15.07 ± 8.71
GDS, mg/dl (mean±SD)	118 ± 101
Urea (mean±SD)	20.96 ± 21.81
Creatinine (mean±SD)	0.96 ± 0.88
CRP (mean±SD)	1.05 ± 0.6
Chest X-ray (n, %)	
Normal	3 (10.7)
Bronchopneumonia	18 (85.7)
Symptoms (n, %)	
Asymptomatic	0 (0)
Light	0 (0)
Moderate	22 (78.6)
Heavy	4 (14.3)
Critical	2 (7.1)
Length of care (mean±SD)	5 ± 4
Maternal output (n, %)	
Life	23 (82)
Die	5 (18)

In this study, the average patient reproductive age is the age of 29 ± 6 years, with good nutritional status, which is indicated by the average size of the upper arm circumference of 26.7 ± 1.2 cm. The average pregnant woman infected with COVID-19 has a good Hb value, 11.7 ± 2.1 g/dl, leukocytes 12800 ± 7128 / L. Pregnant women with COVID-19 infection showed an increase in the value of D-dimer (1447 1072 L/ml), fibrinogen (554 ± 182 mg/dl). We also found that only 1 patient with positive SARS-CoV-2 RT-PCR had a non-reactive antigen swab result. The pregnant woman with COVID-19 infection who came to Haji Adam Malik Hospital showed moderate symptoms were 22 (78.6) patients chest X-rays showed bronchopneumonia in 18 (85.7 %) patients (Table 1), while the average length of stay for pregnant women with COVID-19 infection is 5 4 days, with an average gestational age at termination was 36 1 weeks (Table 1). In this study, it was observed that 5 patients died, and 23 patients were survived and recovered after treatment. Based on our analysis, we did not find any significant differences in characteristics between patients who died and patients who recovered except for upper arm circumference (26.4 ± 1.1 vs 28 ± 0.8; p = 0.006), blood glucose (92 ± 29 vs 234 ± 208; p = 0.035), urea levels (13.45 ± 5.54 vs. 48.4 ± 42.52; p = 0.006), and creatinine (0.66 ± 0.24 vs. 1.69 ± 1.86; p = 0.01). It was found that patients who died from SARS-CoV-2 in pregnancy tended to be older than the survived and recovered groups (33 ± 7 vs 29 ± 6; 0.412) but this was not statistically significant. We also found that the length of stay of patients who died was longer when compared with patients who lived and recovered (6 ± 3 vs. 5 ± 4; 0.101), but we failed to show any statistically significant, albeit trending, the difference in length of stay and longer hospitalization of patients who died. However, in this study, we found that most patients who survived and recovered had chest X-ray bronchopneumonia (83.3%) but did not differ from the group who died (p = 0.794). The reason for this difference may be that the number of cases who died was significantly different from the group who lived and recovered, so this study was not able to prove a significant difference.

### Histopathology of the placenta and N Protein SARS-CoV-2 expression in the placenta

In the histopathological examination of the placenta, we performed macroscopic and microscopic examinations according to the standard protocol. The mean placenta measures 18x17x2.5 cm and weighs 564 g without fetal membranes or an umbilical cord. The umbilical cord showed three vessels and no thrombosis, was 14 cm long, inserted eccentrically, and measured 1.2 cm in diameter. The placental membranes are translucent, complete, and morphologically normal. The fetal surface shows subchorionic fibrin and both surfaces appear normal. In the excised section, no villous lesions were observed.

**Figure 2:**
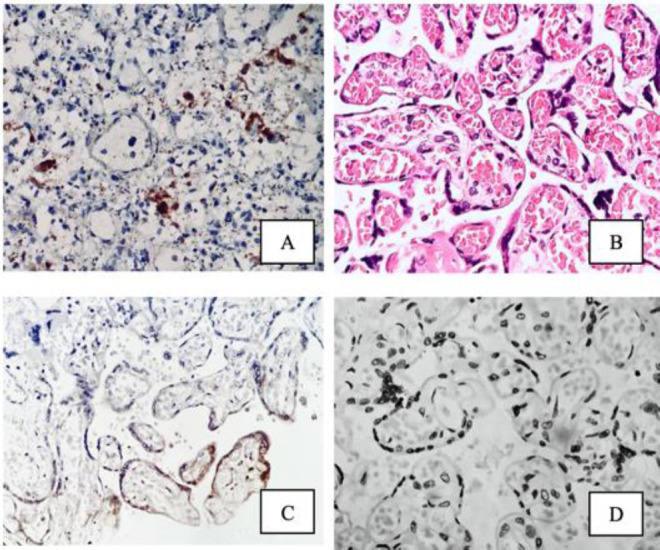
(a) Positive control of IHK rabbit antibody-antigen SARS-CoV-2 (COVID-19) nucleocapsid antibody [HL344] in lung tissue of patients with RT-PCR confirmed COVID-19 infection (400x magnification); (b) Histopathology of placental tissue with hematoxylin and eosin staining (400x magnification); (c) Histopathology of placental tissue from patients with RT-PCR confirmed COVID-19 infection, positive CPI (400x magnification); (d) Histopathology of placental tissue from patients with RT-PCR confirmed COVID-19 infection, negative CPI (400x magnification).

In general, the pathological features shown in the placenta of mothers with COVID-19 infection vary widely. In some cases, we found fibrosis of the villi, with congested blood vessels and thickened vessel walls. We also found features of chronic inflammation and villitis, with lymphocyte infiltration of inflammatory cells. In other cases, we found interstitial bleeding and a knotting appearance of the umbilical cord. The villi showed focal inflammatory infiltrate consisting of macrophages, plasma cells, and numerous lymphocytes (Fig. 2b). In addition, mononuclear cells in the decidual and avascular villi adjacent to the subchorionic thrombohematoma. Maternal vessels did not show features of decidual vasculopathy (both chorioamnionitis and funusitis).

In immunohistochemical staining using rabbit antibody-antigen SARS-CoV-2 (COVID-19) nucleocapsid antibody [HL344], only 2 placental preparations showed SARS-CoV-2 N protein expression, namely in case 13 and case 18, whereas in other placental preparations we did not find any expression of N protein.

**Table 3:** Analysis of Preeclampsia Incidence in SARS-CoV-2 Infection.

Variable	Case 1	Case 2	Case 3	Case 4	Case 5
age (years)	23	38	42	32	29
LILA (cm)	28.79	28.56	28.44	27.58	26.79
Gravida	1	3	3	1	2
parity	0	0	2	0	1
Abortion	0	2	0	0	0
co-morbidities	Eisenmenger syndrome	Preeclampsia	Preeclampsia	Preeclampsia	Preeclampsia
Gestational age at termination (weeks)	30	30	38	28	36
Antigen_SARSCOV2	positive	positive	positive	positive	positive
SpO2 (%)	70%	70%	60%	70%	50%
Ventilator	yes	yes	yes	yes	yes
HB (g/dl)	17.4	12.4	9.5	13.1	13.5
HT (%)	54	35	28.6	43.3	39.5
Leu (/ml)	38410	11390	7150	21510	10210
PLT (/ml)	356000	495000	157000	480000	246000
Neu Abs (%)	33.69	11.21	6.34	20.34	8.94
Lim Abs (%)	2.48	1.18	0.46	0.47	0.99
D-dimer (uL/ml)	903	583	583	1500	1140
Fibrinogen (mg/dl)	328	390	468	529	900
Symptom	Hard	Hard	Critical	Hard	Critical
SGOT	25	26	30	53	19
SGPT	19	13	15	19	23
GDS (mg/dl)	101	267	138	584	82
urea	103	17	19	86	17
Creatinine	5	0.78	0.73	1.18	0.75
CRP	0.7	2.8	1.4	0.7	0.7
Thorax Photo	BP	BP	BP	BP	BP
Length of Treatment	9	9	4	6	3
BBL (grams)	1000	1220	3345	1100	2855
Baby RT-PCR	Negative	Negative	Negative	Negative	Negative
APGAR	2/3	3/4	8/9	0/0	8/9
Placenta Weight (grams)	469	479	526	426	497
Baby Output	Death	Death	Life	OF	life
NBS	15	15	35	10	30
Baby hematology				
HB (g/dl)	9.6	8	12.9	11.3	17.4
HT (%)	28	23.9	36.5	39.1	50.8
Leu (/ml)	15180	7750	11300	45980	9060
PLT (/ml)	43000	165000	314000	139000	8000
Neu Abs (%)	9.94	2.86	8.33	44.85	4.88
Lim Abs (%)	3.98	3.74	1.35	0.46	3.18
Baby Care Length	1	5	12	0	2
SARS-CoV-2 Placental Protein N Expression	Negative	Negative	Negative	Negative	Negative

The two patients who died were aged 38 and 42, which are the ages at high risk for pregnancy. Of the 5 mothers who died, two of them were obese, with LILA 28.79 and 28.56. Two cases were terminated at 30 weeks gestation and one case was terminated at 28 weeks gestation. In the five cases of mothers who died, all of them came with comorbidities. In 4 cases, preeclampsia was found, while one case showed a congenital heart defect which is a contraindication for pregnancy. The five cases showed oxygen saturation in the range of 50-70% with oxygen attached and on a ventilator with various modes. In these five patients, there were 3 cases with infant death outcomes, two babies died after termination, and one patient with IUFD (intrauterine fetal demise). In case 1 and case 4, we found leukocytosis (38,410/ml; 21,510/ml), while the other death cases showed normal leukocyte levels. All cases of death showed elevated levels of D-dimer and fibrinogen. All cases died showing symptoms of COVID-19, 3 of them came with severe symptoms, and 2 cases with critical symptoms. In case 2 and case 4 we found a transient increase in glucose levels (267 mg/ml: 584 mg/dl). In cases 1 and 4, we found elevated levels of urea (103; 86) and creatinine (5,1;18). All cases of death showed bronchopneumonia on chest X-ray with varying lengths of stay (3-9 days). The last two babies who died weighed 1000 grams and 1220 grams, while in the case of KJDK, babies born weighed 1100 grams. In case 1 and case 2, the baby’s APGAR score at birth was 2/3 and 3/4 in case 1 and case 2, we found anemia in newborns with HB levels of 9.6 g/dl, and 8 g/dl. In the infants of case 1 and case 4, we had leukocytosis (15,180/ml; 45,980), which may have been caused by sepsis and resulted in infant death.

## Discussion

Current epidemiological data suggest that COVID-19 can affect pregnant women, but there is too little data that confirms the prognosis in pregnant women. In the current situation, when most countries are fighting the SARS-CoV-2 epidemic, even incomplete data can be useful for planning the protection of infected pregnant women(Khan *et al.*, 2020; Lu *et al.*, 2020).

Gurol-Urganci *et al*. (2021), in their study reported that most pregnant women with COVID-19 infection were aged 30-34 years and most of them were multigravida. Approximately 10.8% of

Xu *et al*. (2020) in their studies reported that the mean gestational age of patients with SARS-CoV-2 infection in pregnancy varied widely, ranging from 26-41 weeks.

Breslin *et al*. (2020), reported from the 7 cases that they treated, 2 cases presented with complaints of uterine contractions, while 5 other cases came with the main complaints of cough, fever, and myalgia. Two cases in the study of Breslin *et al*. (2020), required intensive care unit (ICU) treatment, while the other 2 cases were treated in the usual isolation room and 3 cases were sent home for self-isolation (Breslin *et al.*, 2020). Laboratory results in the study of Breslin *et al*. (2020), showed leukocyte levels between 4500-7400/ml, while platelet levels ranged from 129000-276000/ml (Breslin *et al.*, 2020).

Hu *et al*. (2021) also reported that 4 of the 7 patients they treated with COVID-19 infection in pregnancy showed symptoms of fever without other symptoms such as cough, dyspnea, or diarrhea. In 6 patients, pregnancy termination by cesarean section was carried out and only one patient was given antiviral before termination of pregnancy (Hu *et al.*, 2021).

In the study by Zhou *et al*. (2020) in 9 pregnant women (8 singleton pregnancies, 1 twin pregnancy) with confirmed COVID-19 infection, clinical symptoms were found in 4 cases before delivery, in 2 cases on the day of delivery, in the remainder after delivery (7 cesarean sections, 2 cases natural childbirth). Most of the first symptoms were fever and cough, and 1 patient had diarrhea. Six women gave birth prematurely and 3 at term. Although no newborn was confirmed to be infected, 6 had dyspnea, 2 had a fever, 2 had thrombocytopenia with normal liver function, had an increased heart rate, 1 had vomiting, and 1 had a pneumothorax. One of the newborns died in our study, and termination was carried out at 36 weeks, whereas in the analysis (Table 2) termination in the case group (dead mother) was performed at a lower gestational age of 32 ± 4 weeks, with 2 babies eventually died and 1 baby experienced fetal death in the womb (IUFD) (Zhou *et al.*, 2020).

**Table 2:** Maternal Output.

Variable	Maternal Output		*P-Value*

	Life (23)	Death (5)	
Age, years (mean ± SD)	29 ± 6	33 ± 7	0.305^a^
Body weight, kg (mean ± SD)	56 ± 4	55 ± 3	0.348*
Height, cm (mean ± SD)	158 ± 5	154 ± 4	0.161^a^
Body mass index, kg/m2(mean ± SD)	22.5 ± 1.7	23 ± 2.1	0.816*
Upper arm circumference, cm (mean ± SD)	26.4 ± 1.1	28 ± 0.8	0.006^a^
Gravida (mean ± SD)	2 ± 1	2 ± 1	1*
Parity (mean ± SD)	1 ± 1	1 ± 1	0.483*
Abortion (mean ± SD)	0 ± 1	0 ± 1	0.641*
Gestational age at termination, weeks (mean ± SD)	36 ± 2	32 ± 4	0.071*
SARS-CoV-2 antigen (n, %)			
Not Reactive	1 (4.3)	0 (0)	0.929^c^
reactive	22 (95.7)	5 (100)	
Hb, g/dl (mean ± SD)	11.4 ± 1.8	13.2 ± 2.8	0.247^a^
HT, % (mean ± SD)	34 ± 5	40 ± 10	0.236^a^
Leukocytes, /μL (mean ± SD)	11728 ± 5104	17734 ± 12754	0.355^a^
Platelets, /μL (mean ± SD)	259087 ± 90125	346800 ± 146607	0.258^a^
Absolute Neutrophils, % (mean ± SD)	9.42 ± 4.26	16.1 ± 11.16	0.253^a^
Absolute Lymphocytes, % (mean ± SD)	1.58 ± 0.82	1.12 ± 0.83	0.301^a^
D-Dimer, L /ml (mean ± SD)	1459 ± 1200	1477 ± 741	0.965^a^
Fibrinogen, mg/dl (mean ± SD)	558 ± 179	523 ± 224	0.758^a^
SGOT (mean ± SD)	125.67 ± 349.42	33.5 ± 13.18	0.316*
SGPT (mean ± SD)	54.75 ± 149.71	16.5 ± 3	0.133*
GDS, mg/dl (mean ± SD)	92 ± 29	234 ± 208	0.035*
Urea (mean ± SD)	13.45 ± 5.54	48.4 ± 42.52	0.006*
Creatinine (mean ± SD)	0.66 ± 0.24	1.69 ± 1.86	0.01*
CRP (mean ± SD)	0.91 ± 0.34	1.4 ± 0.99	0.454*
Chest X-ray (n, %)			
Normal	3 (13)	0 (0)	0.794^c^
Bronchopneumonia	20 (87)	5 (100)	
Symptoms (n, %)			
Asymptomatic	0 (0)	0 (0)	
Light	0 (0)	0 (0)	0.745^d^
Moderate	22 (95.7)	0 (0)	
Heavy	1 (4.3)	3 (60)	
Critical	0 (0)	2 (40)	
Length of care (mean ± SD)	5 ± 4	6 ± 3	0.208*

Chen *et al*. (2020) in their study, reported 43.9 women with COVID-19 infection during the third trimester of pregnancy. Although none of the women had preexisting comorbidities such as diabetes or cardiovascular disease, a single case of COVID-19 infection was reported to result in hypertension in pregnancy and preeclampsia. Chen *et al* also reported that the clinical symptoms of COVID-19 were like symptoms in non-pregnant patients, namely: fever (7/9), cough (4/9), muscle aches (3/9), sore throat (2/9), malaise (2/9), gastrointestinal symptoms (1/9), and dyspnea (1/9). Laboratory examinations showed elevated CRP (6/9), lymphopenia (5/9), alanine aminotransferase (ALT), and aspartate aminotransferase (AST) (3/9). Chest CT scan was abnormal in 8 of 9 women. In all cases, pregnancies were terminated by cesarean section, 4 women delivered prematurely, but none were terminated below 36 weeks of gestation (Chen *et al.*, 2020).

Zhang *et al*. (2020) performed a retrospective analysis comparing the outcomes of pregnant women infected with SARS-CoV-2. 16 patients had pneumonia and 45 without pneumonia. Only 1 of the patients with pneumonia had a severe condition. The method of delivery in both groups was a cesarean section, and weeks of gestation were (38.7 ± 1,4) weeks and (37.9 ± 1.6) weeks, with no significant differences found. There was no difference in intraoperative blood loss, neonatal weight loss, and no infected newborn (Zhang *et al.*, n.d.).

According to available data, the effect of COVID-19 on placental histology is divided into two. Some investigators found the presence of COVID-19 in the placenta but argued that no placental histopathological differences were observed, while some studies indicated that some pathological changes occurred. The author tries to further analyze the pathological changes that occur in the placenta in this study. All placentas in this study belonged to subjects from pregnant women who tested positive for COVID-19 and were confirmed by RT-PCR examination

In this study, we found that most placentas showed villitis, although immunohistochemically we only found 2 placentas showing N protein expression of SARS-CoV-2 which indicates that this virus replicates in the placenta, so it is still not clear exactly what causes the occurrence of villitis. However, 41% of lesions initially classified as villitis of unknown etiology (VUE) have been reported in one study to have a viral infectious etiology demonstrated by electron microscopy. Recent data on VUE in live births show that lesions in the villi are usually composed of maternal and fetal macrophages (CD4 and CD8). Ozer *et al*. (2020), in their study also reported that macrophages and CD4-positive T cells predominate in SARS-CoV-infection in the placenta, although an increased CD8-positive cell count is also present (Ozer *et al.*, 2020).

There is an immunological paradox in normal pregnancy, namely a shift from a Th1 to a Th 2 type response. Suppression of cell-mediated and antibody-mediated immune responses by anti-fetal mechanisms confers an advantage in maintaining pregnancy. Cytokines involved in the Th2-mediated immune system include IL-4, IL10, and TGF-β, while anti-inflammatory cytokines IL-2, IL-6, and IL-12 are more dominant in the Th1-6-mediated immune system (Khan *et al.*, 2020).

Grifoni *et al*. (2020), reported that, in cases of SARS-CoV-2 infection in the lungs, specific CD4+ T cells were found in all cases of COVID-19, whereas CD8+ T cells were found in most cases but not in all cases of COVID-19, the immunological reaction triggered by SARS-CoV-2 infection showed increased mobility of several cytokines such as IL-1, IL-6, IL-12, IFN-γ and TNF-α. Based on the study of Grifoni *et al*, we found similar characteristics of cytokines involved in cases of VUE in the placenta due to COVID-19 infection in the report of Ozer *et al.*, (2020). Based on this, the authors conclude that there may be a shift in the immune system in pregnant women with COVID-19 infection, which was initially predominantly mediated by Th2 cells to Th1 cells. This shift in the immune response can certainly harm future fetal outcomes (Grifoni *et al.*, 2020).

In a recent cohort study, maternal COVID-19 infection was found to be significantly associated with fetal vascular thrombosis (Gurol-Urganci *et al.*, 2021; Villar *et al.*, 2021). In addition, VUE sometimes progressed to obliterative fetal vasculopathy. The presence of CD8+ cytotoxic T cells is responsible for the development of the frequently observed fetal placental vasculopathy in VUE as well as the increased apoptosis of intervillous cells. This may explain why we did not find any feature of fetal vascular malperfusion in our case. In addition, obliterative fetal vasculopathy occurs in response to chronic inflammation in VUE when there is multifocal involvement. This may also explain the absence of fetal vascular malperfusion as chronic villitis in our case was low grade. In addition, no umbilical cord abnormalities or maternal hypercoagulability are known to be associated with fetal vascular malperfusion.

The authors conclude that SARS-CoV-2 infection in this study may be associated with VUE resulting from the anti-viral response. Since SARS-CoV-2 is a virus, it will most likely cause inflammation. This is a response of Th1 and Th2, especially CD4-positive lymphocytes that result from the normal tolerance process of pregnancy. The immunogenic activity of these cells is influenced by cytokines which are also increased in SARS-CoV-2 infection. The authors, therefore, hypothesized that the increase in inflammatory cytokines that accompanies SARS-CoV-2 infection may produce VUE lesions. Further studies are needed to prove this hypothesis regarding whether VUE is associated with the antiviral immune response to COVID-19.

## Conclusion

Histological features of the placentas of mothers with COVID-19 infection in our study varied widely. In some cases, we found fibrosis of the villi, with congested blood vessels and thickened vessel walls. We also found features of chronic inflammation and villitis, with inflammatory cell lymphocytes. In other cases, we found interstitial bleeding and a knotting appearance of the umbilical cord. In this study, we found 2 cases with N protein expression of SARS-CoV-2 on placental CPI staining. In our analysis, we did not find any difference in the data on the characteristics of the mother and the outcome of the baby with the expression of the SARS-CoV-2 N protein in the placenta, and all infants were negative on the SARS-CoV-2 RT-PCR examination. In this study, we found 4 cases with preeclampsia, but none of the placentas from these 4 cases showed the expression of the SARS-CoV-2 N protein, so we suspected that SARS-CoV-2 infection could exacerbate the condition of preeclampsia, and not as a cause of the onset of preeclampsia in pregnant women.

### Statement on Conflict of Interest

The authors declare that there is no conflict of interest associated with this study.

**List of Abbreviations Used:**
3CLpro: chymotrypsin-like protease;
ACE2: angiotensin converting enzime 2;
AnCE: Angiotensin-converting enzyme (Ance) gene of Drosophila;
ARDS: acute respiratory distress syndrome;
AT2: Alveolus Type 2;
CD =: Connector Domain;
CD4: Clusster Differentiaion 4;
CD8: Clusster Differentiaion 8;
CH: Central Helix;
COVID-19: Corona Virus Disease 19;
Protein E: protein envelope;
Protein M: protein membrane;
Protein N: protein nukleokapsid;
Protein S: protein spike;
SARS: severe acute respiratory syndrome;
SARS-CoV-2: severe acute respiratory syndrome corona virus disease 2;
TMPRSS2: protease transmembrane serine protease 2.
